# Long term effects of cariprazine add-on therapy in the patients with risperidone induced hyperprolactinemia

**DOI:** 10.1192/j.eurpsy.2025.2072

**Published:** 2025-08-26

**Authors:** J. Dedovic, A. Tomcuk, A. Macic, V. Roganovic, S. Dedovic

**Affiliations:** 1 Addiction Department; 2 Centre for Mental Health Promotion; 3 Management Department, Special Psychiatric Hospital Dobrota Kotor, Kotor; 4 Health Center Budva, Budva, Montenegro

## Abstract

**Introduction:**

The treatment strategies for antipsychotics induced hyperprolactinemia apart from switching to prolactine-sparing antipsychotics, reducing the doses of antipsychotics and adding dopamine agonists such is bromocriptine, include add-on therapy with third generation antipsychotics (aripiprazole, cariprazine)

During the previous years, some studies with adjunctive aripiprazole therapy have been conducted, however the research with cariprazine in this purpose are absent.

**Objectives:**

The main goal of this research is to analyse both short-term and long-term changes in the level of prolactine after adding cariprazine in the long acting risperidone treatment of patients with psychotic disorders.

**Methods:**

Six inpatients threated during the first three months of 2024, that were consecutive diagnosed with hyperprolactinemia induced by long acting risperidone Depo therapy, were included for the participation in this research. For all study participants, the long acting antipsychotics therapy was previously used for more than 3 months and in all of them other possible causes of hyperprolactinemia were excluded by neuroimaging procedures and examinations of consultant endocrinologist. After starting adjunctive therapy with cariprazine in the dose range between 1.5 and 3mg daily, for at least three weeks, while risperidone therapy remained at the same dose, prolactine levels were firstly re-examined. The second control of prolactine levels were done after 6 months.

**Results:**

All patients were diagnosed with ICD 10 categories of psychotic disorders, 2 of them (33.3%) with F20 category (Schizophrenia), 3 of them (50%) with F 29 category (nonspecific psychosis) and 1 of them (16,6%) with F23 category (acute psychosis). On average age of the patients were 37,6 years, 3 of them had male and 3 female sex. Prolactine base values (T0) were between 612 mlU/L and 4051,53 mlU/L (on average 2150,7 mlU/L). After introducing cariprazine adjunctive therapy for at least three weeks, the levels of prolactine (T1) substantially declined in five out of six patients (83,3%). This second values of prolactine were in the range between 306 mlU/L and 2014,4 mlU/L which represents 22,9% to 67% reduction of their initial level (on average 47,34%). In only one study case, the prolactine level has raised from 667,73 to 765,88 (14,7%). The third value of prolactine (T2) were re-examined after the 6 months of introducing the cariprazine treatment and values of prolactine remained at low levels.

**Conclusions:**

The main conclusion of this pilot study can be that cariprazine adjunctive therapy is valuable pharmacological intervention for the treatment of risperidone induced hyperprolactinemia and that these effects sustain over the 6 months period. However, the number of participants in this research is rather small, and for that reason, further investigation at the bigger number of participants are necessary for definite conclusions.

**Disclosure of Interest:**

J. Dedovic Grant / Research support from: In the past I have received several honoraria for lectures organised by Richter Gedeon. However this research has not been paid by any commercial organisation., A. Tomcuk: None Declared, A. Macic Grant / Research support from: In the past I have received several honoraria for lectures organised by Richter Gedeon. However this research has not been paid by any commercial organisation., V. Roganovic: None Declared, S. Dedovic: None Declared

